# An EEG study of detection without localisation in change blindness

**DOI:** 10.1007/s00221-019-05602-2

**Published:** 2019-07-23

**Authors:** Catriona L. Scrivener, Asad Malik, Jade Marsh, Michael Lindner, Etienne B. Roesch

**Affiliations:** Centre for Integrative Neuroscience and Neurodynamics, School of Psychology and Clinical Language Sciences, University of Reading, Reading, UK

**Keywords:** Change blindness, Sensing, Event-related potentials, Awareness

## Abstract

Previous studies of change blindness have suggested a distinction between detection and localisation of changes in a visual scene. Using a simple paradigm with an array of coloured squares, the present study aimed to further investigate differences in event-related potentials (ERPs) between trials in which participants could detect the presence of a colour change but not identify the location of the change (sense trials), versus those where participants could both detect and localise the change (localise trials). Individual differences in performance were controlled for by adjusting the difficulty of the task in real time. Behaviourally, reaction times for sense, blind, and false alarm trials were distinguishable when comparing across levels of participant certainty. In the EEG data, we found no significant differences in the visual awareness negativity ERP, contrary to previous findings. In the N2pc range, both awareness conditions (localise and sense) were significantly different to trials with no change detection (blind trials), suggesting that this ERP is not dependent on explicit awareness. Within the late positivity range, all conditions were significantly different. These results suggest that changes can be ‘sensed’ without knowledge of the location of the changing object, and that participant certainty scores can provide valuable information about the perception of changes in change blindness.

## Introduction

Change blindness is a phenomenon in which changes to a visual scene are often missed (Rensink 2004; Simons and Levin 1997). To manipulate this in an experimental setting, the change blindness paradigm typically consists of two images displayed in quick succession that are interrupted by a blank screen or a distractor image. In some instances, the second image is identical to the first, and in others, some aspects will have changed. Participants are then asked to report if the trial contained a change or not. The complexity of these images varies across paradigms, ranging from coloured rectangles (Koivisto and Revonsuo 2003) and coloured dots (Schankin and Wascher 2007), to facial expressions (Eimer and Mazza 2005), detailed visual scenes (Fernandez-Duque et al. 2003) and household objects (Busch et al. 2010). In all cases, although complete visual information is available, participants often fail to notice or identify changes.

Most versions of the change blindness paradigm ask participants to detect the presence of a change across two image presentations, meaning that trials can only be categorised as one of four types: hit (or see trials), miss (or blind trials), false alarm (FA), or correct rejection (CR), depending on whether the participant reports seeing a change. Several researchers have challenged the traditional view that vision must always be accompanied by a complete conscious visual experience, or the activation of complete internal representation of what we see (Rensink 2004; Fernandez-Duque and Thornton 2000), and subsequently suggested the possibility of further trial divisions in the change blindness paradigm. In an early experiment reported by Rensink (2004), participants were asked to indicate when they ‘thought’ that something had changed in a flicker paradigm, and again when they were certain that they could see the change. In a flicker paradigm, the original image and changed image are presented sequentially until the participant is able to detect the change (Rensink et al. 1997). Trials in which these responses had a time difference greater than 1 second were labelled as trials with a ‘significant duration of sensing’, where the participant suspected a difference but was not confident in their perception of the change. Rensink (2004) termed the ability to detect a change without fully identifying it as sensing, suggesting that this condition is both phenomenologically and perceptually distinct to the traditionally reported see condition.

Several other researchers have explored the possibility of an awareness condition that lies somewhere between the traditional see and blind dichotomy (Fernandez-Duque et al. 2003; Laloyaux et al. 2006; Thornton and Fernandez-Duque 2001; Galpin et al. 2008; Busch et al. 2009; Ball and Busch 2015; Kimura et al. 2008; Hollingworth et al. 2001). For example, Fernandez-Duque and Thornton (2000) found that the location of a change could be identified above chance level even when participants did not report seeing the change itself [but see Mitroff et al. (2002) and Laloyaux et al. (2006) for a discussion of these results]. Further, in Mitroff et al. (2004) participants were able to identify pre- and post-change object stimuli above chance level when they detected a change, as well as when they did not. The presence of a sense condition has, therefore, been suggested as evidence that change blindness may arise from a failure to compare two displays or images, rather than a failure to encode the visual information (Simons and Ambinder 2005; Hollingworth et al. 2001). Further, sense trials may occur when features of a changing object only reach a pre-attentive stage, and are not fully integrated at later stages of visual processing (Galpin et al. 2008; Busch et al. 2009).

Results from change blindness experiments using EEG appear to support this assertion. In previous EEG research, the trial types of see and blind are often distinguishable in an early visual attention component around 200–300 ms after the change onset at contralateral electrode sites, known as the N2pc (Luck and Hillyard 1994; Schankin and Wascher 2007). The presence of an N2pc reflects the allocation of attention towards an attended object (Luck and Ford 1998), and the amplitude is increased for ‘aware’ stimuli (Schankin and Wascher 2007). However, the N2pc also been found for ‘unaware’ stimuli in a masking paradigm, and, therefore, does not necessarily represent conscious awareness of a change (Woodman and Luck 2003). It is, therefore, suggested that the N2pc, in the context of change blindness, reflects processing that is necessary, but not sufficient, to facilitate conscious change detection (Schankin and Wascher 2007; Busch et al. 2009).

There is also evidence that the amplitude of early visual components, such as P1 and N1, may be dependent on the awareness level of the participant during a change detection task, given that larger peaks are identified for stimuli occurring in an attended location (Pourtois et al. 2006; Railo et al. 2011; Luck and Ford 1998). However, not all change blindness EEG studies succeed in replicating this effect (Koivisto and Revonsuo 2010).

In a similar time window to the P1/N1 complex (around 200 ms), the visual awareness negativity (VAN), typically occurring at posterior electrode sites, is thought to indicate detection of a stimulus and be dependent on spatial attention (Koivisto et al. 2008, 2009; Wilenius and Revonsuo 2007). It has been suggested that the VAN is associated with phenomenal visual awareness and is present even when successful identification of a changed object is not achieved (Lamme 2004; Busch et al. 2009).

VAN is often followed by later positive ERP at posterior electrode sites called the late positivity (LP) (Koivisto et al. 2009). This overlaps with the P3 component, also peaking around 400 ms, and can also be referred to as such in the literature (Busch et al. 2009). In comparison to the VAN, the LP is associated with conscious aspects of task processing (Railo et al. 2011), and has been shown to correlate with participants’ confidence in their responses (Eimer and Mazza 2005) .

Several EEG papers have also identified differences between see, sense and blind conditions. In a comparison between trials in which the participants were able to detect a change and identify the object of the change (see), and those where they could detect a change but not name it (sense), Busch et al. (2010) found an increase in amplitude of the VAN. The same effect was found in a later LP ERP at posterior electrodes. However, the N2pc peak was found only when participants could both detect and identify the change, and was not present when participants were change blind, or could not identify the object. The authors concluded that seeing a change is not simply a stronger version of sensing a change, as the N2pc can be found for see trials but not sense trials. This supports the hypothesis of Rensink (2004) that seeing and sensing may be facilitated by separate mechanisms. Other studies have also found differences in ERP amplitudes when comparing see and sense (Fernandez-Duque et al. 2003; Kimura et al. 2008; Busch 2013; Ball and Busch 2015), but the definition of sense trials varies across studies (Mitroff et al. 2002), leading to divergent results.

**Fig. 1:**
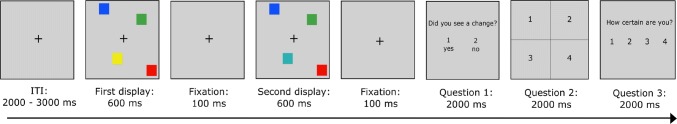
Illustration of the experimental paradigm. The number of squares presented varied from 2 to a maximum of 36. Question 1 asked ‘Did you see a change?’ to which participants could respond ‘Yes’ or ‘No’. Question 2 asked participants to localise the change, based on a grid from top left to bottom right. Question 3 asked how certain participants were of their responses, ranging from ‘1: Very Uncertain’ to ‘4: Very Certain’. If participants responded ‘no change’ to question 1, they were moved straight on to question 3

The main aim of the present study was to compare behavioural and ERP effects for trials in which participants could report the presence of a change but not localise it (sense), versus those in which participants could report and localise the change correctly (localise). Specifically, we divided the visual display into quadrants, and asked participants to select the quadrant in which the change occurred. Our sense condition, therefore, requires registration of the change, but not necessarily knowledge of its location (Mitroff et al. 2002). Further, participants were asked to rate how confident they were in their responses at every trial, to distinguish between trial types (Galpin et al. 2008). We used a simple paradigm with an array of coloured squares (see Fig. 1).

As increased amplitudes in the N2pc and LP have previously been found in the see condition compared to the blind condition, we hypothesised that we would replicate these findings (Railo et al. 2011). Although modulation of P1 amplitudes have been reported in some change detection paradigms (Busch et al. 2009; Pourtois et al. 2006), others report no such effect (Eimer 2000; Turatto et al. 2002; Niedeggen et al. 2001), so our hypothesis was not directed. When comparing localise versus sense trials, we hypothesised that we would find increased amplitudes in the VAN, LP, and N2pc for localise trials (Busch et al. 2010; Fernandez-Duque et al. 2003).

A further aim of the study was to identify if sense trials are behaviourally different to blind or false alarm trials, as others have suggested (Fernandez-Duque et al. 2003; Galpin et al. 2008), or whether they can be explained by explicit mechanisms (Mitroff et al. 2002). If the sense condition (where participants can detect but not localise a change in coloured square) can be explained by participant pressing the incorrect response when they did not see a change, then reaction times for sense trials should be similar to blind trials. Or, if sense can be explained by a liberal response criteria, such that participants report seeing a change despite not being sure, then uncertain sense trials should have similar reaction times to false alarms. Using EEG measures of neural activity, as well as additionally asking participants to rate their confidence at each trial (Galpin et al. 2008), we aimed to distinguish between these distinct types of awareness.

## Materials and methods

### Participants

Twenty subjects (mean ± SD, age = 20 ± 5, 6 left handed, 2 male) with no history of psychiatric or neurological disorders participated in this EEG study. All participants were over the age of 18, had corrected-to-normal vision and were not colour blind (based on self-report). The experiment was approved by the University of Reading ethics committee (UREC: 17/03), and was conducted in accordance with the Declaration of Helsinki (as of 2008). All participants gave informed consent to take part, including consent to share their anonymised data. Three participants were removed from the original sample size of 23 for having less than 200 usable trials after pre-processing (out of a maximum of 250 trials). Trials were classified as unusable if they contained muscle or eye-movement artefacts that could not be removed during pre-processing.

### Stimuli and presentation

Participants were presented with a change blindness task using Psychtoolbox (Kleiner et al. 2007), on a 1920 $\times$ 1080 LCD monitor with a 60 Hz refresh rate. Participants were seated comfortably on an armchair, at approximately 60cm away from the screen, alone, in a quiet room (Faraday cage) with constant dim light. They were asked to fixate on a central fixation cross and identify changes between consecutive displays of coloured squares. These were interrupted by a short fixation display to facilitate the change blindness phenomenon (see Fig. 1 for details on display durations). On change trials, one of the squares changed colour from the first to the second display. On no-change trials, the displays were identical. This was followed by two or three questions, depending on the participant’s response to the first question. Each participant completed 5 blocks of 50 trials, leaving a total of 250 trials. Within these trials, two-thirds contained a change in coloured square (165 trials), and the rest contained no change (85 trials).

Question 1 asked ‘Did you see a change?’ to which participants could respond ‘yes’ or ‘no’ using a keyboard. Question 2 asked participants to localise the change, based on a 2 $\times$ 2 grid from top left to bottom right. Question 3 asked how certain participants were of their responses, ranging from ‘1: Very Uncertain’ to ‘4: Very Certain’. If participants responded ‘no’ change to question 1, they were moved straight to question 3. This decision was made as our hypotheses did not relate to ‘implicit’ change detection, as reported in Fernandez-Duque and Thornton (2000), and removing this question allowed for a greater number of trials within the same period of time. Participants were asked to respond within a limit of 2 s for each question, and trials with any response missing were not included in further analysis (3.6 ± 2.9). Participants made their response on a keyboard, using their index and middle fingers of each hand.

Difficulty was modulated in real time by adding and removing two squares from the display, based on the assumption that more distractors increase task difficulty (Vogel et al. 2005). This was to prevent floor and ceiling performance during the task as a result of individual differences (Luck and Vogel 2013), and optimise for performance rather than to establish specific individual thresholds. Performance over the previous two trials was used to update the current trial; two consecutive correct answers added two squares, two incorrect deducted two squares, and one correct and one incorrect resulted in no change. The decision to increase or decrease the number of squares was made using responses to the localisation question (Q 2), as we were specifically interested in controlling the number of sense and localise trials. The display was divided into a 6 $\times$ 6 grid of possible change locations, meaning that a maximum of 36 squares could be presented during each trial. The location of the change on each trial was random, but the change occurred an equal number of times on the left and right hemifield of the screen. The number of squares always changed by two, to balance the number on the left right hemifields of the screen, and all participants began the experiment with two squares presented. Each block began with the number of squares presented on the last trial of the previous block. As the colour of the squares was not related to our main hypotheses, we used seven default MATLAB colours: blue, cyan, yellow, green, white, red, and magenta (MathWorks, Inc., version 2016b).

### Behavioural analysis

The trials in which a change occurred were divided into three conditions: blind (no change detection), localise (change detection and localisation), and sense (change detection without localisation). Trials in which no change occurred were divided into correct rejection (no change reported) and false alarm (change incorrectly reported). The number of false alarm trials was low, with a mean of 12.45 trials (range 2–33, SD 0.65) and, therefore, EEG analysis comparing false alarm to sense trials was not possible. The percentage of false alarm trials was calculated in relation to the total number of no-change trials, whereas the percentage of sense trials was calculated in relation to the total number of change trials.

Detection accuracy for each participant was calculated based on the percentage of change trials in which they correctly detected a change. Localisation accuracy was calculated as the percentage of correctly detected changes where the localisation was also correct. We also recorded each participant’s mean and maximum difficulty scores, with the maximum referring the highest number of squares that were displayed to them during the experiment.

D’prime was calculated as a measure of participant response bias. This was calculated using the equation $$d = z ($$hit rate$$) - z ($$false alarm rate) (Stanislaw and Todorov 1999), and is defined as the difference between the means of signal and noise distributions, normalised by the variance. Response bias, or criterion, was also calculated, where $$c = -0.5 \times (z($$hit rate$$) + z($$false alarm rate)) (Stanislaw and Todorov 1999). $$c=0$$ indicates no response bias to either ‘yes’ or ‘no’ responses. $$c>0$$ indicates a bias towards ‘no’ responses, with fewer hits and fewer false alarms. $$c<0$$ indicates bias towards ‘yes’, with more hits but also more false alarms. We expected that participants would display a range of response strategies.

One problem faced in identifying a sense condition is that it is difficult to distinguish these trials from false alarm trials, or those where participants press the wrong response key (Simons and Ambinder 2005; Mitroff et al. 2002). Rensink (2004) found that reaction times for sense trials were shorter for change trials than no-change trials, meaning that participants were slower when they were simply making a false alarm. Galpin et al. (2008) also found greater certainty associated with sensing during change trials, compared to false alarms. We, therefore, compared reaction times across awareness conditions, as well as between levels of certainty. As trial numbers were low, ‘very uncertain’ and ‘uncertain’ responses were combined, and ‘certain’ and ‘very certain’ were combined. Each awareness condition, therefore, had two levels of certainty; for example, localise certain and localise uncertain.

### EEG data acquisition

EEG data were recorded with a BrainVision EasyCap (Brain Products), with 64 passive electrodes including an IO channel, arranged according to the 10-10 layout. The reference electrode was placed at FCz and the ground at AFz. Impedance was kept below 10 k$\Omega$ for all the EEG channels, and 5 k$\Omega$ for the IO channel. EEG signals were recorded using BrainVision Recorder (Brain Products, version 1.20) at a sampling rate of 5000 Hz.

### EEG pre-processing

Raw EEG data were pre-processed using BrainVision Analyzer (Brain Products, version 2.1). The data were first downsampled to 500 Hz to reduce computation time, then filtered with a high-pass filter of 0.01 Hz to remove low-frequency drift (Butterworth, second order). A low-pass filter of 50 Hz and a notch filter of 50 Hz were chosen to remove line noise. Independent component analysis (ICA) was used to remove eye-movement artefacts (FastICA). Two components were removed for each participant: one corresponding to eye-blinks and the other to lateralised eye-movements.

Further analysis was completed using EEGLab (Delorme and Makeig 2004). Trials were marked as outliers if any ERP value was greater than three standard deviations from the mean value of that ERP across all trials (using the MATLAB function ‘isoutlier’). Note that we only searched for outliers in the electrodes used for analysis (P07, P08, Cz, Pz, and CPz). Trials marked as containing outliers were excluded from further analysis (3.25 trials per participant ± 2.46), as well as those where a response to any question was not made within the response time (3.60 trials per participant ± 2.94).

Segments were then taken from − 200 to 7000 ms to include the whole trial, and baseline corrected using a mean of the data within − 200 to 0 ms, where 0 ms was the start of the first display of coloured squares (see Fig. 1). We chose the baseline period to be before the first display onset, rather than the second, as we were interested in visual ERPs that occurred in response to both the displays. It has also been suggested that ERPs in response to the first presentation of a stimuli are related to the subsequent perception of change (Pourtois et al. 2006).

### EEG analysis

To identify the peaks of the visually evoked potentials (P1 and N1), a grand average ERP was calculated across all conditions and participants, as advised in Luck and Gaspelin (2017), from electrodes P07 and P08. From here, the peaks of interest were determined by identifying the local maxima/minima of the expected peaks, using the peak detection function in BrainVision Analyzer. The mean value within a window around the peak was used instead of the peak value, as the mean is more robust against noise (Luck 2014). A window of 40 ms around the mean was chosen as the appropriate window for visual ERPs P1 and N1. In relation to the first display onset, the first P1 was identified at 122 ms, and the first N1 at 212 ms. In relation to the second display onset, the second P1 was identified at 114 ms, and the second N1 at 222 ms.

Based on the previous literature (Busch et al. 2010; Tseng et al. 2012; Fernandez-Duque et al. 2003), the N2pc was defined as the mean within 200–400 ms after the second display at occipital electrodes PO7 and PO8. Over central parietal electrodes Cz, CPz and Pz, the VAN was defined within a window of 130–330 ms after the second display, and the LP within a window of 400–600 ms. We used window sizes of 200 ms, defined a priori, in an attempt to be conservative given the large variation within the literature.

To assess how differences between early visual components across detection conditions were reflected at each stimulus presentation, P1 and N1 amplitudes were compared in two separate 2 $\times$ 3 repeated measures ANOVAs, with display (first/second) and awareness (blind/localise/sense) as the independent variables. Differences across hemispheres in the N2pc were analysed with another 2 $\times$ 3 repeated measures ANOVA, with the independent variables of hemisphere (contralateral/ipsilateral) and awareness (blind/localise/sense). Amplitudes of the VAN and the LP were compared in two separate repeated measures ANOVAs with awareness (blind/localise/sense) as the independent variable. Where Mauchly’s test of sphericity indicated that the assumption had been violated, Greenhouse–Geisser correction was used. All post hoc comparisons were two tailed, and corrected for multiple comparisons using false discovery rate, where $q = 0.05$ (Benjamini and Hochberg 1995). Effect sizes are reported as partial eta squared for ANOVA, and repeated measures Hedge’s *g* for *t* tests (Lakens 2013).

To determine if the visual ERPs (P1 and N1) varied as a function of the task difficulty (the number of squares presented per trial) we correlated the single-trial P1 and N1 amplitudes with the number of squares presented at each trial. To determine if the LP amplitude varied with participant confidence, as previously suggested (Eimer and Mazza 2005), single-trial LP values were correlated with participant confidence ratings. For single-trial analysis, time courses were constructed for each participant from the single-trial values of each ERP, at each channel (7 ERPs, 64 channels, 20 participants). Note that midline electrodes were not included in N2pc analysis, as the N2pc values were calculated as the difference between ipsilateral and contralateral amplitudes, which by definition is not meaningful for electrodes on the midline. Each single-trial value was calculated as the mean amplitude within the pre-defined ERP window at each trial. These values were baseline corrected by subtracting the mean of the trial from which they were selected. *P* values were corrected for multiple comparisons using false discovery rate where $q = 0.05$ (Benjamini and Hochberg 1995).

## Behavioural results

### Accuracy and difficulty

Accuracy for question 1, in which participants had to identify a change, had a mean of 49% (range 32–73%, SD 13). Accuracy for question 2, in which participants had to localise the change, had a mean of 70% (55–87%, 8). The mean difficulty level given to each participant was 14 squares (10–18, 3), with the mean maximum difficulty experienced by each participant at 26 squares (20–36, 4). D’prime scores had a mean of 0.61 (0.74–1.64, 0.27). In a one-sample *t* test, D’prime was significantly different from zero, suggesting that participants were able to distinguish between change and no-change trials $t(19) = 19.293, p < 0.001$. Two participants had a negative criterion, meaning that they had a response bias towards false alarms. All other participants had positive criterion, indicating a conservative response strategy ($0.60 \pm 0.42$).

Mean difficulty did not correlate with detection accuracy ($r = -0.022, p = 0.928$), location accuracy ($r = 0.136, p = 0.566$), or d’prime ($r = -0.229, p = 0.332$), suggesting that the difficulty of the task did not influence task performance. Maximum difficulty also did not correlate with detection accuracy ($r = 0.067, p = 0.779$), location accuracy ($r = -\;0.077, p = 0.748$), or d’prime ($r = -\;0.148, p = 535$).

### Comparison of sense and false alarm trials

The percentage of false alarm trials ($14.64\% \pm 11.35$) was lower than the percentage of sense trials ($30.31\% \pm 8.02$) $t(19) = -\;7.107, p < 0.001, g_{{\text {rm}}} = 1.48$, suggesting that sense trials occurred more often than participants made false alarms. However, the percentage of false alarms was positively correlated with the percentage of sense trials ($r = 0.527, p = 0.017$). Therefore, participants with a more liberal response strategy who made more false alarms, also had more sense trials.

Reaction times for sense and false alarm trials were compared, to determine if sense trials were different to trials where the participant incorrectly reported a change during a no-change trial. Reaction times for all sense trials ($0.744 \pm 0.149$ s), regardless of certainty, were not significantly different to false alarm trials ($0.778 \pm 0.179$ s), $t(19) = -\;1.229, p = 0.234, g_{{\text {rm}}} = 0.193$. However, sense certain trials ($0.619 \pm 0.133$ s) were significantly faster than false alarm trials, $t(19) = -\;4.741, p < 0.001, g_{{\text {rm}}} = 0.939$. Therefore, when participants were certain that a change occurred, they responded more quickly than when they were simply making a false alarm.

Reaction times for sense certain trials ($0.619 \pm 0.133$ s) were also significantly faster than false alarm uncertain trials ($0.817 \pm 0.211$ s), $t(19) = -\;4.510, p < 0.001, g_{{\text {rm}}} = 1.081$. However, this may be explained by the general finding that, across all conditions, certain trials ($0.628 s \pm 0.142$) were faster than uncertain trials ($0.849 \pm 0.129$ s), $(t(19) = -\;7.831, p < 0.001, g_{{\text {rm}}} = 1.563)$

### Comparison of sense and blind trials

Reaction times for sense trials ($0.744 \pm 0.149$ s) were not significantly different to blind trials ($0.731 \pm 0.176$ s), $t(19) = -\;0.285, p = 779, g_{{\text {rm}}} = 0.082$. However, reaction times for sense uncertain trials ($0.801 \pm 0.189$ s) were significantly slower than blind trials, $(t(19) = 4.424, p < 0.001, g_{{\text {rm}}} = 0.373)$. Therefore, on trials where the participant did not see the change (blind), they responded more quickly than when they suspected a change but could not provide additional information about it (sense).

Comparatively, reaction times for sense certain trials ($0.619 \pm 0.133$ s) were significantly faster than blind uncertain trials ($0.860 \pm 0.231$ s), $(t(19) = 4.424, p < 0.001, g_{{\text {rm}}} = 1.224)$, which again may be explained by the fact that uncertain trials were slower over all conditions.

### Comparison of blind trials and no-change trials

Out of the 20 participants included in the analysis, 15 were slower to respond when they were blind to the change, compared to no-change trials (75%). This difference in reaction times was not significant when comparing all no-change trials ($0.704 \pm 0.167$ s) to blind trials ($0.731 \pm 0.176$ s), $(t(19) = -2.084, p = 0.051, g_{{\text {rm}}} = 0.143)$. However, blind uncertain trials ($0.860 \pm 0.231$ s) were significantly slower than no-change trials ($0.704 \pm 0.167$ s), $(t(19) = 3.637, p = 0.002, g_{{\text {rm}}} = 0.718)$. Therefore, despite being blind to the change, the presence of a change in the display increased reaction times, particularly for trials where the participant was uncertain.

## EEG results

### Single-trial correlations

The purpose of this analysis was to check whether single-trial ERPs varied as a function of difficulty, i.e. the number of squares presented on the screen during each trial. After correcting for multiple comparisons using FDR correction ($q = 0.05$), no significant correlations were found.

The second analysis was to test whether single-trial ERPs varied with the confidence ratings of the participants. Several researchers have suggested that ERPs, particularly those in later time windows such as the LP, may be more influenced by participant confidence in their response than by the level of conscious awareness (Koivisto and Revonsuo 2003; Eimer and Mazza 2005). None of the tests were significant, with all $p > 0.34$. This result suggests that confidence ratings were not directly correlated with single-trial ERP amplitudes.

### P1 and N1

**Fig. 2:**
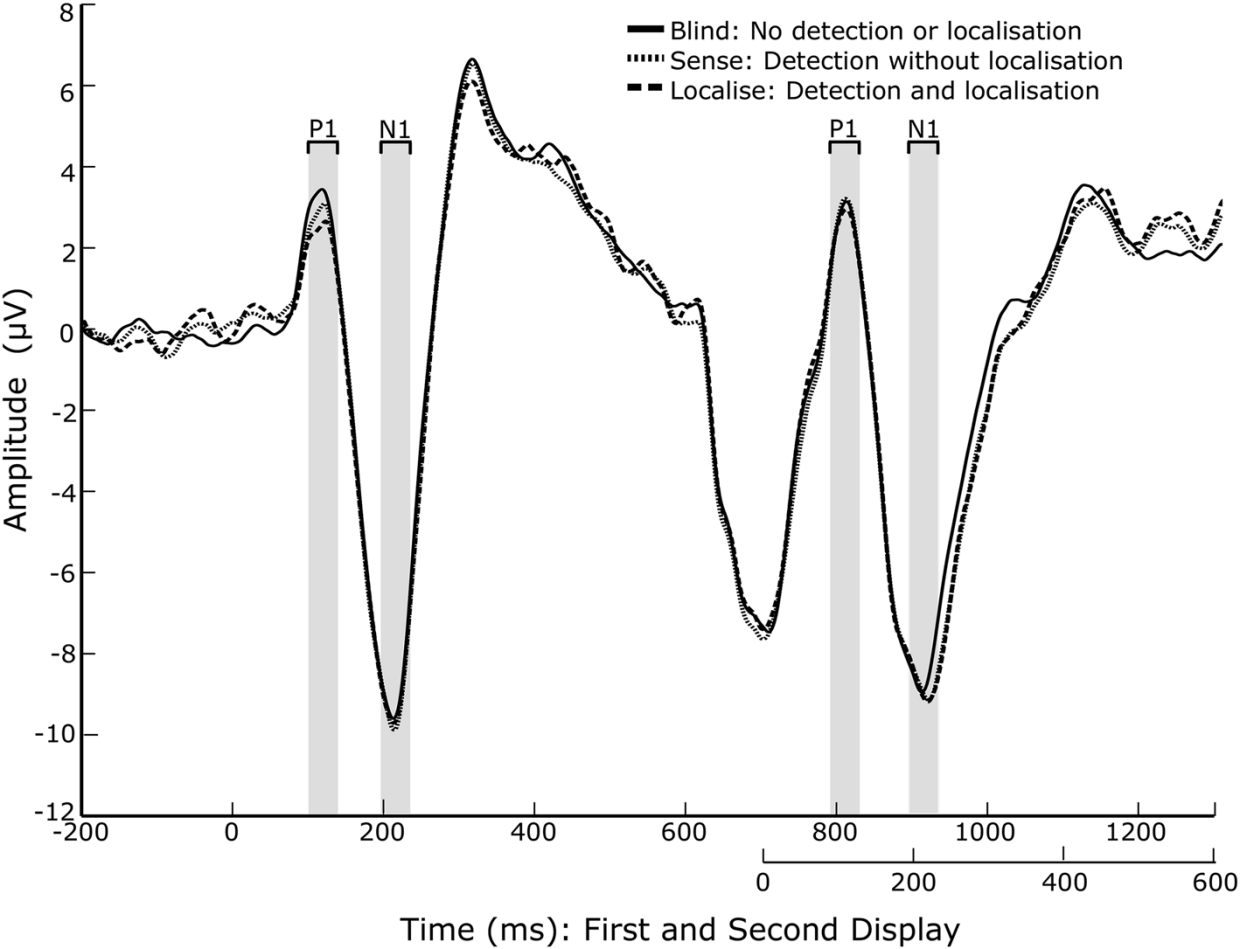
ERP plot showing the mean of electrodes PO7 and PO8, for each awareness condition. Condition means for the values within the shaded time windows were used for ERP analysis

Overall, no significant differences were found between the three awareness conditions for either the P1 or N1 (Fig. 2). For P1 amplitudes, the main effect of awareness was not significant, $F(1.473,19) = 1.117, p = 0.338$, $\eta ^{2} = 0.056$. The main effect of display was also not significant, $F(1,19) = 0.355, p =0.558, \eta ^{2} = 0.018$, nor was the interaction between awareness and display, $F(1.80,34.35) = 0.307, p = 0.305$, $\eta ^{2} = 0.060$.

For the N1, the main effect of awareness was not significant, $F(1.36,19) = 3.534, p = 0.060$, $\eta ^{2} = 0.157$. The main effect of display was also not significant, $F(1,19) = 0.209, p = 0.653, \eta ^{2} = 0.011$, nor was the interaction between awareness and display, $F(1.87,35.61) = 0.377, p = 0.675$, $\eta ^{2} = 0.019$.

### N2pc

**Fig. 3:**
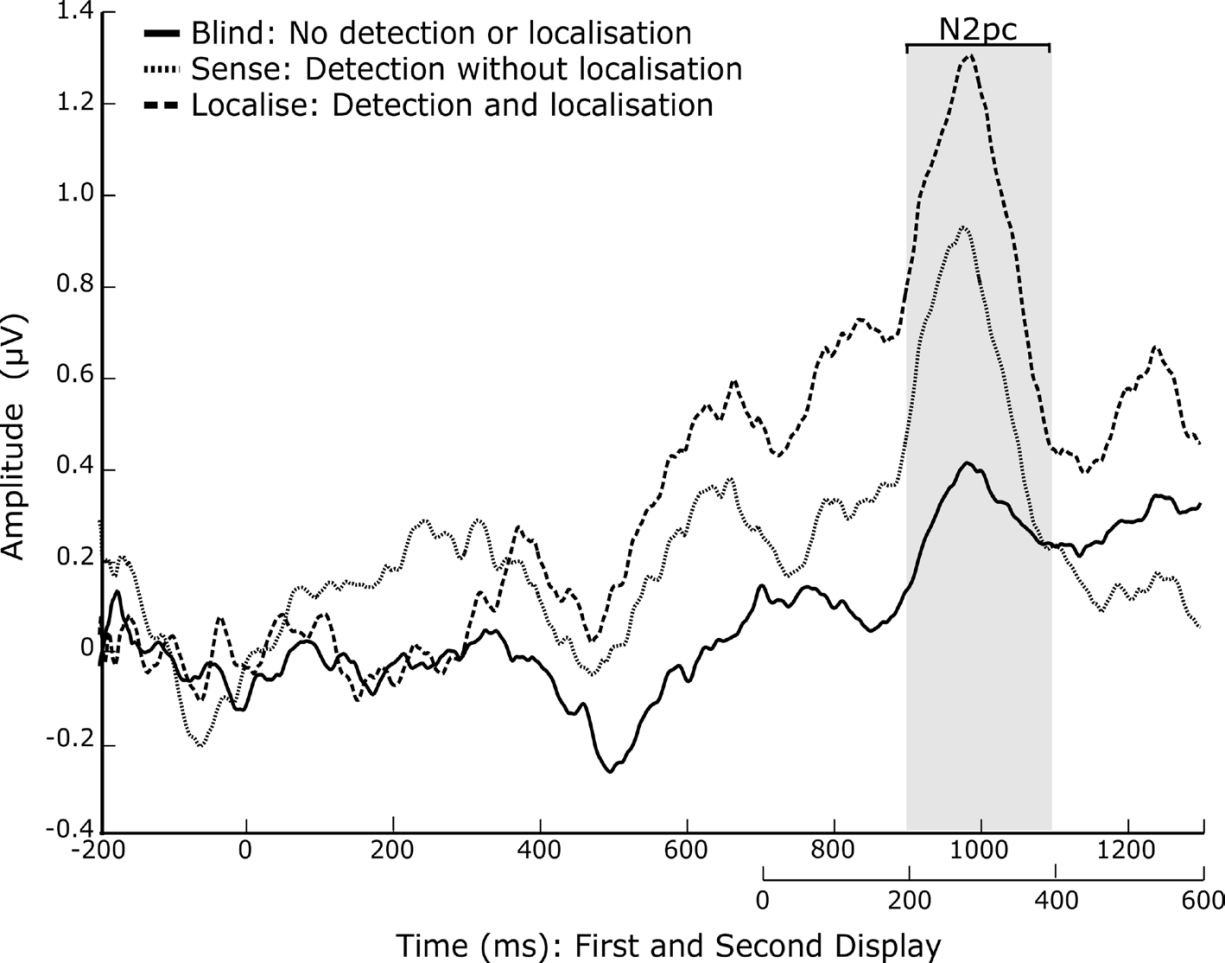
ERP plot showing the mean of electrodes PO7 and PO8, for each awareness condition. Asymmetry was calculated by subtracting contralateral from ipsilateral waveforms. Condition means for the values within the shaded time window (200–400 ms after the second display) were used for N2pc analysis

In line with our hypothesis, there was a significant main effect of awareness on N2pc amplitudes, $$F(2,18) = 4.043, p = 0.026$$, $$\eta ^{2} = 0.175$$ (Fig. 3). There was also a significant main effect of hemisphere, $$F(1,19) = 4.594, p = 0.045$$, $$\eta ^{2} = 0.195$$, with a greater negativity in the contralateral hemisphere ($$-2.89$$$$\pm 3.97$$$$\mu V$$) than the ipsilateral ($$-2.33$$$$\pm 4.26$$$$\mu V$$). The interaction was not significant, $$F(2,18) = 1.048, p = 0.361$$, $$\eta ^{2} = 0.052$$.

Post hoc pairwise comparisons across awareness levels with a FDR-corrected threshold of $$p = 0.03$$ showed that blind ($$-2.055$$$$\pm 1.23$$$$\mu V$$) had a significantly smaller N2pc amplitude than localise localise, ($$-2.941$$$$\pm 1.80$$$$\mu V$$), $$t(19) = 2.340, p = 0.030, g_{{\text {rm}}} = 0.197$$, and sense ($$-2.847$$$$\pm 1.19$$$$\mu V$$), $$t(19) = 2.525, p = 0.021, g_{{\text {rm}}} = 0.181$$. However, sense and localise were not significantly different, $$t(19) = -0.283, p = 0.780, g_{{\text {rm}}} = 0.022$$.

### Visual awareness negativity (VAN)

**Fig. 4:**
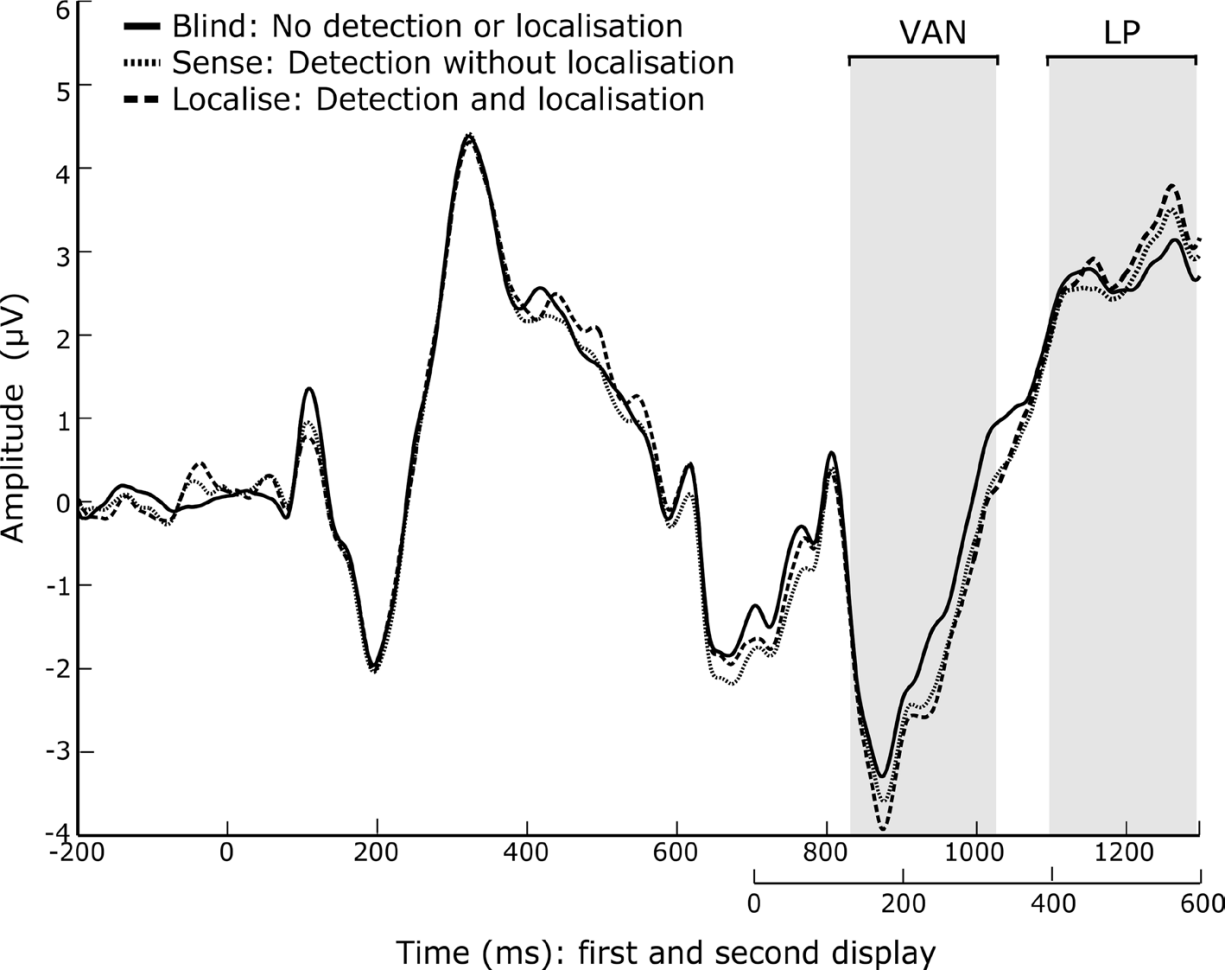
ERP plot showing a mean of electrodes Cz, CPz, and Pz, for each awareness condition. Condition means for the values within the shaded time window were used for ERP analysis. The first shaded area was used for the visual awareness negativity (130–330 ms after the second stimulus), and the second shaded area was used for the late positivity (400–600 ms)

Confirming our hypothesis, there was a significant main effect of awareness on the VAN (Fig. 4), $$F(1.374,18)=3.931, p = 0.046$$, $$\eta ^{2} = 0.171$$. However, in post hoc pairwise comparisons across awareness levels with a FDR-corrected threshold of *p* = 0.04, blind ($$-1.474$$$$\pm 2.52$$$$\mu V$$) was not significantly different to localise ($$-2.167$$$$\pm 3.09$$$$\mu V$$ ), $$t(19) = 2.158, p = 0.044, g_{{\text {rm}}} = 0.217$$, or sense ($$-1.961$$$$\pm 1.92$$$$\mu V$$), $$t(19) = 1.950, p = 0.066, g_{{\text {rm}}} = 0.161$$. Localise and sense were also not significantly different, $$t(19) = 1.235, p = 0.232, g_{{\text {rm}}} = 0.062$$.

### Late positivity (LP)

In support of our hypothesis, there was a significant main effect of awareness on LP amplitudes (Fig. 4), $$F(1.355,8) = 7.000, p = 0.008$$, $$\eta ^{2} = 0.269$$. In post hoc pairwise comparisons across awareness levels with a FDR-corrected threshold of p = 0.048, blind (2.931 $$\pm 2.02$$$$\mu V$$) was significantly smaller in amplitude to both localise (3.905 $$\pm 2.53$$$$\mu V$$ ), $$t(19) = -\;3.094, p = .006, g_{{\text {rm}}} = 0.383$$, and sense (3.591 $$\pm 2.40$$$$\mu V$$), $$t(19) = -2.193, p = 0.041, g_{{\text {rm}}} = 0.275$$. Localise was also significantly greater in amplitude than sense, $$t(19) = 2.110, p = 0.048, g_{{\text {rm}}} = 0.118$$.

## Discussion

The main aim of this change blindness experiment was to distinguish between trials in which participants could both detect and localise a change in coloured square (localise), versus those in which they could only detect it (sense), or not detect it at all (blind). We found significant differences between blind trials and both sense and localise trials in the N2pc ERP. We also found that sense and localise were significantly different in the late LP window. Behaviourally, reaction time results allowed us to distinguish sense trials from false alarm and blind trials, when taking participant certainty into account. Overall, our results suggest that the sense condition may be distinguishable from the traditional see condition, and that utilising participant confidence is a valuable method to distinguish between levels of awareness in change blindness.

### EEG

Our results indicated a difference between sense and localise trials within the LP range, which were significantly different to each other, as well as to blind. An increased late positivity for change detected trials versus change blind trials is the most commonly reported finding within the EEG literature, and all of the papers considered in the review by Koivisto and Revonsuo (2010) report this finding. This may be due to the relatively large size of this ERP, peaking anywhere between 300 and 700 ms after a change stimulus and across large time windows.

While the earlier negativity, VAN, is typically thought to be associated with phenomenal consciousness, the later positivity is linked to access consciousness and greater subject report ability. The repeated finding that the LP can be significantly reduced by specific stimuli, such as non-targets and repeated stimuli, suggests that it is not a direct correlate of visual awareness (Koivisto and Revonsuo 2010). Instead, it is generally thought to reflect higher level or fully conscious aspects of task processing (Railo et al. 2011; Koivisto and Revonsuo 2003). It has also be shown that the LP correlates with confidence in participant responses (Eimer and Mazza 2005). However, when correlating single-trial LP amplitudes with confidence ratings, we did not find a significant effect.

The majority of change blindness papers listed by Koivisto and Revonsuo (2010) reported enhanced negativity in the N1–N2 range (with the exception of Fernandez-Duque et al. 2003; Niedeggen et al. 2001). Busch et al. (2010) found that an N2pc was evoked only when the change was fully identified, and not in the sense or blind conditions. Based on this, they draw the conclusion that for sense trials, the change did not induce a shift in attention towards the location of the change and, therefore, the features of the change were not available for further recognition. This is based on the assumption that the N2pc represents the allocation of attention towards the object of interest, which is supported by a number of previous studies (Luck and Ford 1998).

Contrary to this, we found that both awareness conditions were significantly different to blind trials, indicating a shift in the allocation of attention for all identified changes, regardless of subsequent success/failure to localise. It may also be that sense trials elicited a shift in attention to the correct hemifield of change (and, therefore, subsequently an N2pc), but that it was not specific enough to determine whether the change occurred in the upper or lower field within that hemifield. Woodman and Luck (2003) also identified an N2pc for ‘unaware’ stimuli which were masked by object substitution masking, suggesting that the N2pc does not necessarily represent conscious awareness of changes (Woodman and Luck 2003). It is suggested, however, that the amplitude is increased for ‘aware’ stimuli (Schankin and Wascher 2007), which our findings support.

Other studies have reported a larger N2pc for more attention-demanding tasks (Luck and Hillyard 1994). It was, therefore, a concern before analysis that sense trials would occur more often when the task was more difficult and, therefore, that the N2pc would be larger for this condition as a result of uneven trial distribution. We found the opposite, however, with a smaller N2pc in the sense condition compared to the localise condition. We also found no significant correlation with the number of sense trials and the difficulty of task given to the participant, suggesting that the trial distribution was even enough to avoid this confound.

Although there was a main effect of awareness within the VAN at central parietal sites, the corrected post hoc tests were not significant, and only localise was significantly different to blind using an uncorrected threshold ($p = 0.044$). In comparison, (Busch et al. 2010) were able to identify a VAN for their sense condition, compared to blind. The VAN is thought to be dependent on spatial attention, and requires both the location and identity of an object to be stored such that it is available for conscious report (Koivisto et al. 2008). As participants were not able to identify the location of change in our sense condition, this may explain the lack of significant VAN ERP. In another study (Koivisto et al. 2008), VAN was found to be reduced when participants were asked to keep their eyes fixated at the centre of the screen. This was the case in this experiment, which may also have contributed to the lack of significant finding within the VAN window.

Unlike previous findings from Pourtois et al., the amplitude of the P1 during the first stimuli display was not influenced by the level of awareness (Pourtois et al. 2006). In fact, no significant modulations of awareness were identified within either of the visual ERPs, P1 and N1, across either display, which fails to support previous findings that P1 amplitude during a visual display varies with attention (Wilenius and Revonsuo 2007) and identification of changes (Mathewson et al. 2009). One possible reason for this could be that the number of squares varied across trials, unlike other experiments where the number was fixed (Pourtois et al. 2006) and, therefore, possibly driven by inter-individual differences in performance. However, when correlating single-trial P1 and N1 amplitudes with difficulty across time, no significant correlations were found, after correcting for multiple comparisons. This suggests that the amount of squares presented during each trial had no direct influence on the amplitude of the P1 and N1 and, therefore, that it did not create an obvious confound in the data.

In a review of the ERP correlates of visual awareness, Koivisto and Revonsuo (2010) list a number of change blindness EEG studies that also failed to detect modulation of an early P1 peak (Eimer 2000; Koivisto and Revonsuo 2003; Fernandez-Duque et al. 2003; Schankin and Wascher 2007; Turatto et al. 2002; Niedeggen et al. 2001), compared to two studies which did (Busch et al. 2010; Pourtois et al. 2006). One criticism of the change blindness paradigm is that success relies on the participant paying attention to the first visual display, for the change to be integrated into the short-term memory and the change detected (Simons and Levin 1997). Attention levels, and perhaps ERPs, in response to the first display, may, therefore, have a large influence on the success of the following trial. We did not find any electrophysiological evidence for this occurring, as the amplitude of the P1 and N1 during the first visual display did not correlate with subsequent ERPs, or with performance. It may be, however, that this effect presented itself in a section of the EEG that was not analysed, or that the effect was not strong enough to detect across participants, some of whom may have been more vigilant than others.

The relationship between attention and awareness in change blindness is complex, and we did not attempt to explicitly dissociate the two in our paradigm. In fact, Koivisto and Revonsuo (2010) argue that the change blindness paradigm is not optimal for investigating the relationship between attention and awareness, as change detection is reliant on memory and, therefore, also on attention (given that attention facilitates working memory). It is very possible that attention directed towards a particular stimuli or region of the display increased the probability of detection, and enabled participants to localise the change successfully. As previously found, attention may be necessary but not sufficient for change detection; changes outside of the focus of attention are often missed, but change blindness can also occur for attended items (Levin and Simons 1997; O’Regan et al. 2000; Chetverikov et al. 2018).

In an attempt to define the independent roles of attention and awareness, Lamme (2004) hypothesised that attention does not determine which stimuli reach a conscious state, but facilitates explicit report of these stimuli. While a large amount of visual input reaches the point where conscious awareness could be achieved, this vulnerable visual experience is short-lived without accompanying attention. Conscious stimuli that are not attended to and, therefore, cannot be explicitly reported only achieve ‘phenomenal awareness’. This is defined as a non-cognitive form of seeing, independent of attention, that can contain information about many items in a visual scene (Lamme 2003, 2004). Similarities can, therefore, be drawn between phenomenal awareness and the sense condition in our experiment, where participants could not successfully report the location of a change. In contrast, stimuli that benefit from the protective mechanism of attention enter ‘access awareness’, and can be explicitly reported. It should also be noted that, within this framework, unconscious stimuli can never be reported, even if attended to.

### Behavioural

One explanation for the presence of a sense condition in change blindness is that it reflects a liberal response criteria, such that participants report seeing a change even though they were not certain that it occurred (Simons and Ambinder 2005). In other words, they make a ‘false alarm’ during change trials. If this is the case, then these trials may be similar in number to false alarm trials, where participants incorrectly report a change for identical displays where they could not have seen a change. We found that participants had a significantly higher percentage of sense trials than false alarm trials, suggesting that sense trials occurred more often. This finding cannot be explained by the fact that more trials contained a change, as the percentages were calculated in relation to the total number of change/no-change trials, respectively.

However, we also found a significant correlation between the percentage of sense and false alarm trials, suggesting that participants with a more liberal response strategy were more likely to report the presence of a change when they were not completely sure where the change occurred. To further compare sense and false alarm trials, we also examined reaction times. Although all sense trials combined were not significantly different to false alarms, sense certain trials were significantly faster. Therefore, sense trials where the participant was certain that they saw something change may be distinguishable from simple false alarms.

Another explanation for the sense condition is that it contains trials for which the participant mistakenly reported a change, even though they were not aware of it. In this case, reaction times for sense trials should be similar to those for blind trials, particularly those where participants were uncertain of their responses. We found that sense uncertain trials were significantly slower than blind trials, suggesting that participants took longer to respond to trials where they suspected that something had changed, but were uncertain.

Previous studies have also reported that participants responded ‘no change’ more quickly for no-change trials, compared to change trials (Williams and Simons 2000; Mitroff et al. 2002). The participant’s response is the same in both trial types, but the presence of a change is different. This suggests that even when they fail to detect the change in a change trial, they take longer to respond. We, therefore, compared reaction times for no-change trials and blind trials. Out of the 20 participants, 15 were slower to respond when they were blind to the change, compared to no-change trials (75%), which is higher than the 68% reported by Williams and Simons (2000). Although no significant differences were found between all blind and no-change trials, blind uncertain trials were significantly slower. It is possible that in blind certain trials, no information about the change is registered by the participant and, therefore, reaction times are similar to no-change trials. However, in blind uncertain trials, some information may be available to the participant, leading to slower reaction times, but not enough for them to be confident to report the change.

As the average accuracy for question 1 (yes/no) was roughly 50% across participants, change trials were fairly equally divided into see (all trials where a change was correctly identified) and blind conditions. Within the see trials, accuracy for question 2 (‘where did the change occur?’) was roughly 70%, leaving more trials in the localise condition than the sense condition.

Unfortunately, the number of false alarm trials was low, meaning that a comparison of false alarms trials in the EEG data was not possible. Within the sense trials, there was also a low number of ‘certain’ trials, meaning that dividing the awareness conditions into certain/uncertain for EEG analysis was also not possible. Future experiments could focus on obtaining higher trial numbers, which would hopefully facilitate this analysis. However, the very nature of the sense condition means that participants are unlikely to be ‘certain’ during many of the trials.

We defined the difficulty of the task as the number of squares that were presented to the participant during each trial. Participants ranged in the difficulty within which they could perform the task with similar accuracy. The maximum difficulty ranged from 10 to 36, with only one participant reaching the highest possible level. The fact that the difficulty measures, such as maximum difficulty and mean difficulty, were not correlated with accuracy or d’prime, suggests that the difficulty modulation managed to control for individual differences in ability across participants. However, despite the difficulty modulation, the range of accuracy demonstrated by the participants was large (32–73%). Future studies could benefit from a more sophisticated measure of trial-by-trial adaptation, to further balance the number trials within each condition and participant.

### Conclusions

Overall, the main aim of this experiment was to identify neural differences between full and partial awareness of colour changes, while controlling for individual differences in performance. Behaviourally, reaction time results allowed us to distinguish sense trials from false alarm and blind trials, when taking participant certainty into account. For EEG data in the N2pc range, localise and sense were both significantly different to blind trials, but not significantly different from each other. In comparison, within the LP range, all conditions were significantly different, indicating that the difference between levels of awareness was represented in this late potential. Overall, our results suggest that the sense condition may be distinguishable from the traditional see condition, and that utilising participant confidence is a valuable method to distinguish between levels of awareness in change blindness.

## Data Availability

The raw and pre-processed data can be found on the Open Science Framework: https://osf.io/thdva

## References

[CR1] Ball F, Busch NA (2015). Change detection on a hunch: pre-attentive vision allows sensing of unique feature changes. Atten Percept Psychophys.

[CR2] Benjamini Y, Hochberg Y (1995). Controlling the false discovery rate: a practical and powerful approach to multiple testing. J R Stat Soc Ser B (Methodol).

[CR3] Busch NA (2013). The fate of object memory traces under change detection and change blindness. Brain Res.

[CR4] Busch NA, Fründ I, Herrmann CS (2009). Electrophysiological evidence for different types of change detection and change blindness. J Cogn Neurosci.

[CR5] Busch NA, Dürschmid S, Herrmann CS (2010). ERP effects of change localization, change identification, and change blindness. NeuroReport.

[CR6] Chetverikov A, Kuvaldina M, MacInnes WJ, Jóhannesson ÓI, Kristjánsson Á (2018). Implicit processing during change blindness revealed with mouse-contingent and gaze-contingent displays. Atten Percept Psychophys.

[CR7] Delorme A, Makeig S (2004). EEGLAB: an open source toolbox for analysis of single-trial EEG dynamics including independent component analysis. J Neurosci Methods.

[CR8] Eimer M (2000). Effects of face inversion on the structural encoding and recognition of faces: evidence from event-related brain potentials. Cogn Brain Res.

[CR9] Eimer M, Mazza V (2005). Electrophysiological correlates of change detection. Psychophysiology.

[CR10] Fernandez-Duque D, Thornton IM (2000). Change detection without awareness: do explicit reports underestimate the representation of change in the visual system?. Vis Cogn.

[CR11] Fernandez-Duque D, Grossi G, Thornton IM, Neville HJ (2003). Representation of change: separate electrophysiological markers of attention, awareness, and implicit processing. J Cogn Neurosci.

[CR12] Galpin A, Underwood G, Chapman P (2008). Sensing without seeing in comparative visual search. Conscious Cogn.

[CR13] Hollingworth A, Williams CC, Henderson JM (2001). To see and remember: visually specific information is retained in memory from previously attended objects in natural scenes. Psychon Bull Rev.

[CR14] Kimura M, Katayama J, Ohira H (2003). Event-related brain potential evidence for implicit change detection: a replication of Fernandez-Duque, et al (2003). Neurosci Lett.

[CR15] Kleiner M, Brainard D, Pelli D, Ingling A, Murray R, Broussard C (2007). What’s new in psychtoolbox-3?. Perception.

[CR16] Koivisto M, Revonsuo A (2003). An ERP study of change detection, change blindness, and visual awareness. Psychophysiology.

[CR17] Koivisto M, Revonsuo A (2010). Event-related brain potential correlates of visual awareness. Neurosci Biobehav Rev.

[CR18] Koivisto M, Lähteenmäki M, Sørensen TA, Vangkilde S, Overgaard M, Revonsuo A (2008). The earliest electrophysiological correlate of visual awareness?. Brain Cogn.

[CR19] Koivisto M, Kainulainen P, Revonsuo A (2009). The relationship between awareness and attention: evidence from ERP responses. Neuropsychologia.

[CR20] Lakens D (2013) Calculating and reporting effect sizes to facilitate cumulative science: a practical primer for t-tests and ANOVAs. Front Psychol. 10.3389/fpsyg.2013.00863 10.3389/fpsyg.2013.00863 PMC3840331 24324449

[CR21] Laloyaux C, Destrebecqz A, Cleeremans A (2006). Implicit change identification: a replication of Fernandez-Duque and Thornton (2003). J Exp Psychol Hum Percept Perform.

[CR22] Lamme V (2004). Separate neural definitions of visual consciousness and visual attention; a case for phenomenal awareness. Neural Netw.

[CR23] Lamme VA (2003). Why visual attention and awareness are different. Trends Cogn Sci.

[CR24] Levin DT, Simons DJ (1997). Failure to detect changes to attended objects in motion pictures. Psychon Bull Rev.

[CR25] Luck SJ (2014). An introduction to the event-related potential technique.

[CR26] Luck SJ, Ford MA (1998). On the role of selective attention in visual perception. Proc Natl Acad Sci.

[CR27] Luck SJ, Gaspelin N (2017). How to get statistically significant effects in any ERP experiment (and why you shouldn’t). Psychophysiology.

[CR28] Luck SJ, Hillyard SA (1994). Spatial filtering during visual search: evidence from human electrophysiology. J Exp Psychol Hum Percept Perform.

[CR29] Luck SJ, Vogel EK (2013). Visual working memory capacity: from psychophysics and neurobiology to individual differences. Trends Cogn Sci.

[CR30] Mathewson KE, Gratton G, Fabiani M, Beck DM, Ro T (2009). To see or not to see: prestimulus phase predicts visual awareness. J Neurosci.

[CR31] Mitroff SR, Simons DJ, Franconeri SL (2002). The siren song of implicit change detection. J Exp Psychol Hum Percept Perform.

[CR600] Mitroff SR, Simons DJ, Levin DT (2004). Nothing compares 2 views: Change blindness can occur despite preserved access to the changed information. Percept Psychophys.

[CR32] Niedeggen M, Wichmann P, Stoerig P (2001). Change blindness and time to consciousness. Eur J Neurosci.

[CR33] O’Regan JK, Deubel H, Clark JJ, Rensink RA (2000). Picture changes during blinks: looking without seeing and seeing without looking. Vis Cogn.

[CR34] Pourtois G, De Pretto M, Hauert CA, Vuilleumier P (2006). Time course of brain activity during change blindness and change awareness: performance is predicted by neural events before change onset. J Cogn Neurosci.

[CR35] Railo H, Koivisto M, Revonsuo A (2011). Tracking the processes behind conscious perception: a review of event-related potential correlates of visual consciousness. Conscious Cogn.

[CR36] Rensink RA (2004). Visual sensing without seeing. Psychol Sci.

[CR37] Rensink RA, O’Regan JK, Clark JJ (1997). To see or not to see: the need for attention to perceive changes in scenes. Psychol Sci.

[CR38] Schankin A, Wascher E (2007). Electrophysiological correlates of stimulus processing in change blindness. Exp Brain Res.

[CR39] Simons DJ, Ambinder MS (2005). Change blindness: theory and consequences. Curr Dir Psychol Sci.

[CR40] Simons DJ, Levin DT (1997). Change blindness. Trends Cogn Sci.

[CR41] Stanislaw H, Todorov N (1999). Calculation of signal detection theory measures. Behav Res Methods Instrum Comput.

[CR42] Thornton I, Fernandez-Duque D (2001). An implicit measure of undetected change. Spat Vis.

[CR43] Tseng P, Hsu TY, Chang CF, Tzeng OJL, Hung DL, Muggleton NG, Walsh V, Liang WK, Cheng Sk, Juan CH (2012). Unleashing potential: transcranial direct current stimulation over the right posterior parietal cortex improves change detection in low-performing individuals. J Neurosci.

[CR44] Turatto M, Angrilli A, Mazza V, Umilta C, Driver J (2002). Looking without seeing the background change: electrophysiological correlates of change detection versus change blindness. Cognition.

[CR45] Vogel EK, McCollough AW, Machizawa MG (2005). Neural measures reveal individual differences in controlling access to working memory. Nature.

[CR46] Wilenius ME, Revonsuo AT (2007). Timing of the earliest ERP correlate of visual awareness. Psychophysiology.

[CR47] Williams P, Simons DJ (2000). Detecting changes in novel, complex three-dimensional objects. Vis Cogn.

[CR48] Woodman GF, Luck SJ (2003). Dissociations among attention, perception, and awareness during object-substitution masking. Psychol Sci.

